# The development, validation and application of remote blood sample collection in telehealth programmes

**DOI:** 10.1177/1357633X221093434

**Published:** 2022-05-10

**Authors:** Albert Koulman, Kirsten L Rennie, Damon Parkington, Carina SB Tyrrell, Michael Catt, Effrossyni Gkrania-Klotsas, Nicholas J Wareham

**Affiliations:** ∗ Albert Koulman and Kirsten Rennie contributed equally to this paper; 1 MRC Epidemiology Unit, 12204 University of Cambridge School of Clinical Medicine, Institute of Metabolic Science, Cambridge Biomedical Campus, Cambridge, UK; 2 Department of Infectious Diseases, 2153 Cambridge University Hospital NHS Trust, Cambridge Biomedical Campus, Cambridge, UK

**Keywords:** Home telecare, pathology, blood sampling, self-care, telehealth

## Abstract

**Introduction:**

The ability to collect blood samples remotely without the involvement of healthcare professionals is a key element of future telehealth applications. We developed and validated the application of the Drawbridge OneDraw device for use at home for blood sample collection. The device was then applied in a large population-based remote monitoring study to assess changes in SARS-CoV-2 IgG antibody levels.

**Methods:**

We tested: (1) feasibility of participants using the device at home without a healthcare professional on the upper arm and thigh sites (2) stability of the dried blood sample collected remotely (3) participant acceptability of the device compared with finger-prick and venous blood samples and the validity of SARS-CoV-2 virus antibody measurement versus venous blood sample (4) application to the Fenland COVID-19 study in which 4023 participants at 3 timepoints across 6 months.

**Results:**

Participant acceptability was high, with a significantly lower median perceived pain score and 76% of participants preferring the OneDraw device over the other blood collection methods. There was high level of agreement in SARS-CoV-2 virus antibody results with venous blood samples in 120 participants (Cohen's kappa 0.68 (95% CI 0.56, 0.83). In the Fenland COVID-19 study, 92% of participants returned a sample at baseline (3702/4023), 89% at 3 months (3492/3918) and 93% at 6 months (3453/3731), with almost all samples received successfully processed (99.9%).

**Discussion:**

The OneDraw device enables a standardised blood sample collection at home by participants themselves. Due to its ease-of-use and acceptability the OneDraw device is particularly useful in telehealth approaches where multiple samples need to be collected.

## Introduction

The COVID-19 pandemic has accelerated interest in developing and implementing telehealth approaches in healthcare and health research.^1–3^ In contrast to the considerable focus on electronic means of communicating with patients and research participants,^4,5^ the development of methods for remote blood sample collection has received relatively little attention. The need for further development of remote sampling approaches was highlighted in the early phases of the pandemic, when opportunities for face-to-face phlebotomy became severely restricted and blood collection by the participant or a family/household member was the only viable option during that period.

Most previous approaches for at home patient blood collection have been based on finger-prick methods to obtain capillary blood, which is spotted on filter paper, similar collection devices or as liquid whole blood sample and have either been conducted by visiting healthcare workers or have been self-administered. Although rarely reported, these methods have been challenged by technical issues related to sample standardisation and by low levels of patient acceptability related to pain. The challenges of patient compliance are accentuated in situations in which repeated blood sampling is required.

To inform a remote monitoring study of changes in SARS-CoV-2 IgG antibody levels within a population (the Fenland COVID-19 study), we developed and validated an approach for at home, repeated blood sample collection, without the need for visits by a healthcare professional (HCP).

To determine whether the OneDraw device could be used to obtain a standardised and adequate dried blood sample, we tested: (1) the feasibility of participants using the device following written and video instructions without a HCP (2) the stability of the blood sample collected at home and sent by postal service to the laboratory compared with a venous blood sample taken by a HCP in clinical conditions and (3) the acceptability of blood collection using the device compared with finger-prick and venepuncture blood collection. The inability to obtain a remote blood sample can obstruct the further development of telehealth approaches, which have made huge advances in development and acceptance due to the COVID-19 pandemic.^4^ We then assessed the validity of the SARS-CoV-2 virus antibody result from the capillary OneDraw blood sample compared with the antibody result using the venous blood draw. Finally, we tested the real-world practicality of this approach in the Fenland COVID-19 study in which 4023 participants were asked to complete blood sample collection at 3 time points across 6 months.

## Methods

A full and detailed description of the methods and experimental design is in the supplementary information.

### OneDraw device

**Figure 1.**
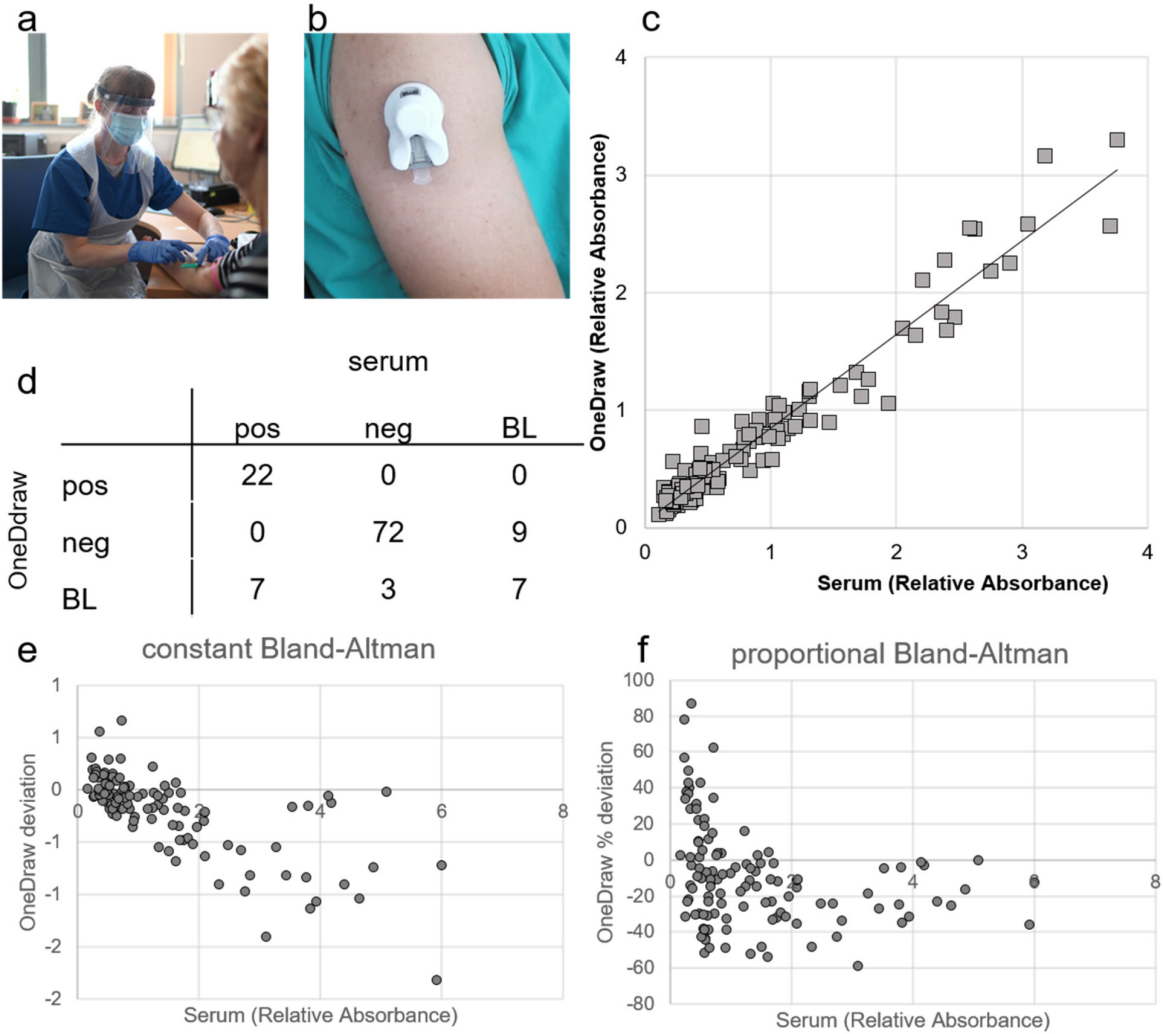
Validation study of blood sample collected by OneDraw device versus venous serum sample. a) Venous blood collection by trained phlebotomist in appropriate personal protection; b) OneDraw device that allows blood collection at home; c) Pearson correlation between SARS-CoV-2 IgG antibody ELISA cut-off ratios from venous serum samples versus dried blood sample from OneDraw device (R^2^ = 0.94; *n* = 120); d) confusion matrix of the classification of the results (*n* = 120, pos = positive (cut-off ratio > 1.1), neg = negative (cut-off ratio <0.8), BL = borderline (cut-off ratio <1.1 & >0.8); e) Constant Bland-Altman plot and f) proportional Bland-Altman plot showing the deviation of the SARS-CoV-2 IgG antibody ELISA results for from dried blood samples collected with the OneDraw device (*n* = 120).

The Drawbridge OneDraw device is licensed for use by HCPs to collect samples for the measurement of HbA1c (FDA 510k)^6^ and CE marked^7^ (Figure 1(a) and (b)). We applied for a dispensation from the UK Medicines and Healthcare products Regulatory Agency, to change the CE licensed use to allow the use of the device at home by a non-healthcare professional for blood sampling. We developed instructions for use by those who do not have assistance (Supplementary Figure 1) and produced online instruction videos (https://www.mrc-epid.cam.ac.uk/research/studies/fenland-covid19/information-for-participants/information-for-all-participants/onedraw-videos/). For blood collection, the OneDraw device is placed on the lateral upper arm or thigh and approximately 150 µL of blood is collected onto two paper strips (8 × 22mm), which sit in a removable cartridge. The resulting blood sample is very similar to a dried blood spot.

### Feasibility study

We assessed if the OneDraw device could be used to obtain a standardised and adequate dried blood sample in the participant's home without the help of an HCP from two body sites. Participants (*n* = 41) were asked to (1) use one of the devices on their upper arm with someone in their household to help them apply the device and (2) self-apply the second device on their thigh. To test the stability of the dried blood spot (DBS) sample after a period of time in transit, participants sent the samples back in the postal service in two separate envelopes to our testing lab using the envelopes provided. Participants were asked to submit feedback on the clarity of the instructions for use and to complete a perceived pain score.^8^

A subgroup of 21 participants additionally came to the clinical facility for standardised blood collection by a trained research team member using three methods in a randomised order; (1) OneDraw device on the upper arm (2) finger-prick and (3) blood sample from venepuncture.

### Validation study

Participants (*n* = 120) were recruited from a population which had previously been tested for SARS-CoV-2 IgG antibodies and we compared antibody test results from blood samples taken using (1) the OneDraw device from the upper arm (2) finger-prick and (3) blood sample from venepuncture, all undertaken at the clinical facility by a trained research team member.

### Fenland COVID-19 study

The primary objective of the Fenland COVID-19 study was to assess SARS-CoV-2 antibody status from the blood samples collected at 0, 3 and 6 months after initial recruitment in July 2020 in participants from an ongoing population-based cohort study in Eastern England. In the initial recruitment period of this study, it was not viable for participants to visit a clinical research facility or for a HCP to visit the home to take a blood sample. We therefore needed to use a home-based blood collection method, such as the OneDraw device. Participants were recruited from the existing Fenland cohort study*.* Participants were asked to complete questionnaires on their health, diet, physical activity and body weight at regular intervals during the study (Supplementary Figure 2).

#### Laboratory analysis

We developed the extraction method using 1.8 mL of phosphate-buffered saline (PBS) overnight for one OneDraw strip and the resulting extract was analysed for SARS-CoV-2 serology,^9^ using a commercial enzyme linked-immunosorbent assay (ELISA), which targets two viral antigens for better sensitivity and specificity: NP (Nucleocapsid protein) and S2 (domain 2 spike protein) from SARS-CoV-2 (Omega diagnostics, UK). In some cases, samples were partially filled and therefore visually graded (Supplementary Figure 3(a)). The grading was used to enable the extraction proportional to a full strip.

### Data analysis

Wilcoxon signed-rank test was used to assess within-person pain score values. Chi-square tests were used to assess differences in categorical variables and ANOVA for continuous variables. The comparability of COVID-19 antibody test results between sample types was assessed using Cohen's kappa coefficient and the agreement using Pearson correlations and Bland Altman plots (expressed as constant and proportional).

### Studies and ethics

Ethics approval for the feasibility study was granted by the University of Cambridge Human Biology Research Ethics Committee and for the validation study by the HRA and Health and Care Research Wales (North West - Haydock Research Ethics Committee). Ethics approval for the Fenland COVID-19 study was granted by HRA (Southwest Cornwall and Plymouth Research Ethics committee).

## Results

### Feasibility of using device for remote blood collection without a HCP

In the sample of 41 adults, 92% of participants (36/39) were able to collect a blood sample successfully at home from the arm and 90% from the thigh (37/41).

### Suitability of dried blood sample for SARS-CoV-2 virus antibody measurement

Repeat analysis of the same samples showed that 1–3 months of storage at −70 °C or at room temperature had no impact on the IgG measurement (Supplementary Figure 4(a)). From the 21 volunteers, two were classified as positive for SARS-CoV-2 virus antibodies and 19 were classified as negative. The quantitative antibody results, expressed as the ELISA cut-off ratio, from blood samples from the OneDraw device were highly correlated with results from the standard serum samples (R^2^ = 0.93) (Supplementary Figure 4(b)).

### Acceptability of different blood collection methods

The median perceived pain scores for use of the OneDraw device at home by participants or a family/household member were 2 for both the upper arm and thigh site respectively, on a scale from 1 (low) to 10 (high) (Supplementary Table 1). The median pain score was significantly lower (*p* < 0.001) for the OneDraw device administered by the researcher on the upper arm site (2 (interquartile range, IQR1-2) than for either the finger-prick (3 (IQR2-4)) or venepuncture blood collection method (3 (IQR 2-4)) (Supplementary Table 2). Overall, 76% of participants preferred the OneDraw device with 0% indicating that the finger-prick blood collection was their preferred modality, and 24% expressing a preference for standard venepuncture blood collection (Supplementary Table 2). Although we tested the modality preference including the option of venepuncture, in the context of a fully remote blood testing deployment without HCP involvement, this option would, by definition, not be available.

**Table 1.** Completion rates of blood sample collection in Fenland COVID-19 study at 0, 3 and 6 months.

Timepoint (month)	Devices posted *N*	Number of participants requiring replacement device, *N* (%)*	Blood samples returned, *N* (%)*	Participants withdrew at timepoint ^#^	Blood sample insufficient to be processed	Valid antibody result *
0	4023	431 (10.7%)	3710 (92.2%)	85	8	3702 (92.0%)
3	3918	508 (13.0%)	3495 (89.2%)	40	3	3492 (89.1%)
6	3731	271 (7.3%)	3456 (92.6%)	16	3	3453 (92.5%)

*Percentage of devices posted.

^#^ Participants withdrew after device posted and did not return sample.

### Validity of SARS-CoV-2 virus antibody results in samples taken by different blood collection methods

The correlation between the quantitative ELISA cut-off results derived from the analysis of the extraction from the OneDraw device strips and those from the analysis of the corresponding samples from the venepuncture was R^2^ = 0.97 (Figure 1(c)). Applying the established manufacturer cut-offs for defining seropositivity, we found complete agreement in the classification of definitive positive and negative results between different sample types. However, there were differences in the classification of samples classified as borderline by either approach (Figure 1(d)). Overall, the results from the samples collected by the OneDraw device showed a high level of agreement with serum separated from venous blood (Cohen's kappa 0.68 (95% CI 0.56, 0.83)) (Figure 1(d)). There was no overall bias in the quantitative read-out comparing results from the OneDraw device with those from serum (Figure 1(e) and (f)).

The results were similar for the standardised finger-prick samples, with blood spotted on paper and air-dried (Supplementary Figure 5). Results from dried blood spot samples collected from a finger-prick also showed also a high level of agreement with those from serum (Cohen's kappa 0.74 (95% CI 0.60, 0.87)).

### Evaluation of real-world practical utility

Overall, 32.5% (*n* = 4030) of those invited consented to take part in the Fenland COVID-19 study. Those who consented to take part were more likely to be female, to be in the highest socioeconomic (SES) category, to have a university degree and less likely to live in a deprived area than the whole cohort.

Overall, 92% of participants returned a sample at baseline (3702/4023), 89% at 3 months (3492/3918) and 93% at 6 months (3453/3731) (Table 1). There were no differences in socio-demographic characteristics between those who returned a blood sample at baseline versus those who did not (Supplementary Table 3). At each time point, some participants were unable to collect a blood sample with the OneDraw device on the first occasion and needed to be sent a replacement device. Second devices were required by 10.7% of people (*n* = 431) at baseline, 13.0% at 3 months (*n* = 508) and 7.3% at 6 months (*n* = 271) (Table 1). A small number of participants (1.1% at baseline, 1.0% at 3 months and 0.2% at 6 months) required a second replacement device (Supplementary Table 4). The number of participants requiring replacement devices repeatedly throughout the study was low; 12.2% of those participants who needed replacements at the 3-month time point had needed replacements at baseline (62/508), and 12.9% of participants at 6 months had needed replacements at the 3-month time point (35/271), respectively. We were able to process almost all (99.9%) of the samples received (Table 1), even though the paper strips were not always completely filled with blood (Supplementary Figure 3(a)). Of all samples received, 7.6% were partially filled, most of these were almost full and fewer than 1.6% of all the samples were less than two-thirds full (Supplementary Figure 3(b)).

**Figure 2.**
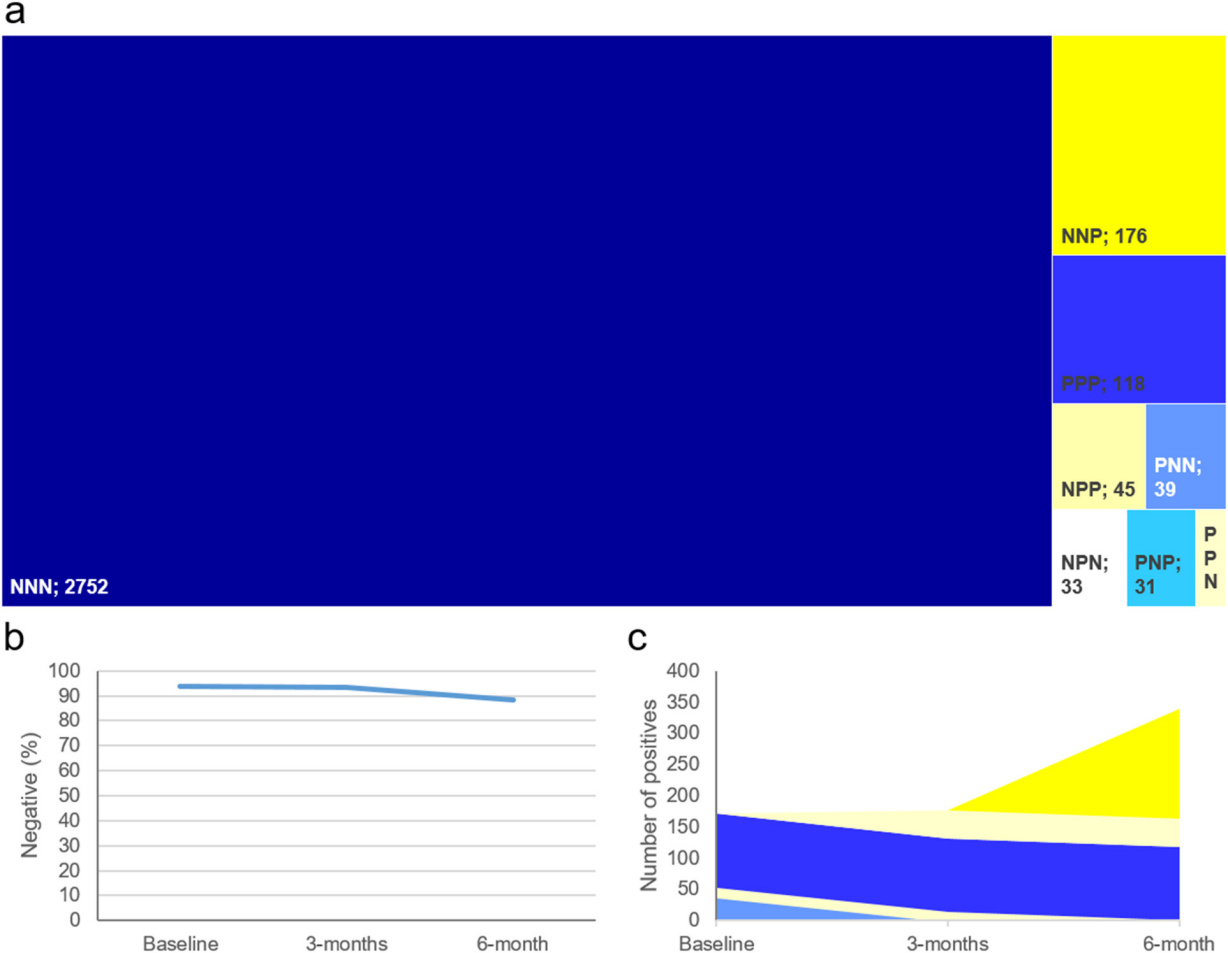
The number of positive cases and percentage of non-positive cases in the group who completed all three sample time points (*n* = 3208). a) Treeplot divided by pattern of positivity/negativity across the three time points (position of the three letters denote time points, P = positive, N = Negative); b) Percentage of participants who did not have a positive test; c) Stacked area plot of how participant converted from negative to positive and vice versa.

In total, 3208 participants provided samples in which COVID-19 antibody status could be determined at each time point (Figure 2(a)). Baseline samples were taken in the summer of 2020, after the first wave of COVID-19 infections in the East of England. The 7-day average case rate was 0.5 per 100,000 on 27 June 2020.^10^ At the baseline 6.3% were positive for SARS-CoV-2 IgG antibodies (Figure 2(b)), which remained constant at the 3-month time point but increased sharply to 11.5% at 6 months. This coincides with the peak and tail of the second wave in the East of England when the 7-day average case rate rose to 16.5 per 100,000 on 27 November 2020, reaching a maximum of 113.7 per 100,000 on 1 January 2021.

## Discussion

The COVID-19 pandemic has accelerated the development of remote methods for delivery of all aspects of preventive and therapeutic health care including the collection of blood samples in ways that do not require attendance at hospital or home visits by HCPs. The results from our work show that technology-driven advances in remote sample collection can facilitate the collection of repeated blood samples at scale in a way that is acceptable to participants and produces valid results. Of the alternative methods that are possible for remote blood taking at home by participants, we have shown that the OneDraw device is much more acceptable to participants than a traditional finger-prick test. Acceptability is a major consideration when repeated measurement is necessary in situations like monitoring of average control of hyperglycaemia by periodic estimation of HbA1c in people with diabetes, for example. The OneDraw device is licensed for use by HCPs to collect samples for the measurement of HbA1c and has been shown to be acceptable by patients for that clinical application.^11^ In research settings, acceptability impacts on initial response rates and is likely to have a major influence on re-measurement rates as in the population cohort study described in this paper.

The development and validation work described in this paper was undertaken to enable the remote collection of blood samples to determine the sero-prevalence of SARS-CoV-2 IgG antibodies at intervals during the study period. When we initiated the study, there were no published results on the use of dried blood samples for SARS-CoV-2 IgG antibody assays, although there were studies that had shown that it was possible to use extractions from dried blood spot for IgG assays for measles, rubella and hepatitis.^12,13^ Subsequently, two studies have reported that it is possible to use dried blood spots with SARS-CoV-2 IgG antibody assays.^14,15^ However, both of these reports are proof-of-principle studies with the dried blood samples being taken by standard finger-prick sampling only. It is not possible to directly compare the acceptability of the finger-prick method used in the previously published study in Sweden compared to the OneDraw device used in our study. In the Swedish study, blood sample kits were posted out to 1000 randomly selected individuals on one occasion rather than on repeated occasions in consented participants as in this study. Furthermore, of these randomly selected individuals, 55% returned a sample and 44% provided a sample that was also suitable for analysis. Our validation study showed that there was no difference in performance in measuring SARS-CoV-2 IgG antibody status between either finger-prick based or OneDraw dried blood samples compared to measurement using serum samples. Kits for finger-prick-based dried blood spot collection cost significantly less than the OneDraw device and can be cost-effective in large-scale studies where return rates are not crucial or when only a single sample per participant is required. For settings in which samples are required at multiple time points, the OneDraw device has considerable advantages.

Devices like OneDraw are easy to use; the vast majority of the participants in this study were able to successfully collect a useable standardised blood sample and post the sample back to the laboratory for analysis. Overall, the effectiveness of collecting blood samples at home using the OneDraw device is high and markedly superior to that of dried blood spots collected from finger pricks^16^; the major reason is that blood transfer onto paper was automated and there was no dependence on the participants’ ability to correctly spot blood onto paper. Where participants were unable to collect samples correctly on the first occasion, this was due to issues such as the creation of an insufficient vacuum by the device or trigger buttons becoming hard to depress, both of which are device-level technological issues that lessened as this study progressed.

There are currently very few clinically approved methods for dried blood spot samples, and they are only routinely used for screening for in-born errors of metabolism. Methods available for plasma or serum will need further development to make these suitable for OneDraw samples. The extraction method developed for the IgG measurement yields enough volume for a range of assays and the OneDraw device could be used to measure a range of factors from the same sample. The remote blood collection will always involve the transport of the blood sample from the participant to the laboratory. Dried blood is a relatively stable environment for many analytes, but not all. It will always be necessary to test the stability of the analyte in the dried blood matrix and during transit, which in this study showed to be of no concern. There are other limitations to the use of such devices in real-world settings, many of which are soluble by future technical developments. Currently, the liquid phase extraction process in the lab involves an element of manual removal of the paper strip from the sleeve in the laboratory, which is slower than an automated process. In addition, the sleeve is coded with patient identifiers but not the paper strip itself. As there is a process of manual removal of the paper strip from the barcoded sleeve, which is then placed into a barcoded tube, there is a possibility of human error. Not all samples that were returned were completely saturated with blood. However, the proportion of samples graded as being incompletely filled (7.6%) was substantially lower than that reported for studies using dried blood spot cards even when administered by trained interviewers, with “one quarter” of the samples having small spots affecting measurement results.^17,18^ However, due to the lack of alternatives, very few studies have assessed the effectiveness of at home participant blood collection in telehealth studies. A manual grading was necessary to assess the amount of blood on the strip, but it should be possible in the future to use imaging software to scan the strip and determine the amount of blood on the strip; a process already in use for standard dried blood spots.^19^ If this were coupled with automation of removal and handling of the sample, the processing of samples would be enhanced and accelerated and thus could become high throughput.

In conclusion, our work shows that it is possible to obtain blood samples from participants remotely with very high efficiency and with a minimal loss in participation at scale. We have deployed a sampling strategy that is significantly more acceptable to participants than finger-prick based dried blood spot sampling, with no loss of analytical quality. We have demonstrated the practical utility of this approach in a large study with high population retention that monitored COVID-19 sero-prevalence over time using a validated assay. This approach has considerable promise as a way of collecting blood samples for other measurements in contexts where remote monitoring is required as part of a move to greater telehealth implementation.

## Supplemental Material

sj-pdf-1-jtt-10.1177_1357633X221093434 - Supplemental material for The development, validation and application of remote blood sample collection in telehealth programmes Supplemental material, sj-pdf-1-jtt-10.1177_1357633X221093434 for The development, validation and application of remote blood sample collection in telehealth programmes by Albert Koulman, Kirsten L Rennie, Damon Parkington, Carina SB Tyrrell, Michael Catt, Effrossyni Gkrania-Klotsas, and Nicholas J Wareham in Journal of Telemedicine and Telecare
