# Supplementary material for: The development, validation and application of remote blood sample collection in telehealth programmes
Source: J Telemed Telecare. 2022 May 10;30(4):731–8. doi: 10.1177/1357633X221093434 (PMC11027437; doi:10.1177/1357633X221093434)
Supplement: sj-pdf-1-jtt-10.1177_1357633X221093434 - Supplemental material for The development, validation and application of remote blood sample collection in telehealth programmes [file sj-pdf-1-jtt-10.1177_1357633X221093434.pdf]

Supplementary information

The development, validation and application of remote blood sample collection in telehealth programmes

Albert Koulman[1]*, Kirsten L Rennie[1]*, Damon Parkington[1], Carina Tyrrell[1], Michael Catt[1], Effrossyni Gkrania-Klotsas[1, 2], Nick J Wareham[1]

* Albert Koulman and Kirsten Rennie contributed equally to this paper

[1]MRC Epidemiology Unit, University of Cambridge School of Clinical Medicine, Institute of Metabolic Science, Cambridge Biomedical Campus, CB2 0QQ, United Kingdom

[2]Department of Infectious Diseases, Cambridge University Hospital NHS Trust, Cambridge Biomedical Campus, CB2 0QQ, United Kingdom

Full methods

Supplementary tables:

Suppl. Table 1: Pain scores and success rate of blood collection using the OneDraw device on the upper arm and thigh

Suppl. Table 2: Comparison of blood collection using OneDraw device with other blood draw methods: pain score and acceptability

Suppl Table 3: Comparison of socio-demographic characteristics of Fenland COVID-19 study participants with valid antibody result with those who did not complete blood sample at each timepoint (0, 3 and 6 months)

Suppl Table 4: Number of replacement OneDraw devices sent

Supplementary figures:

Suppl. Fig 1a: Instructions for use for blood collection form upper arm using OneDraw device

Suppl. Fig 1b: Instructions for use for blood collection form thigh using OneDraw device

Suppl Fig 2: Fenland-COVID study design

Suppl Fig 3. Partially filled strips

Suppl Fig 4: Correlation between SARS-CoV-2 antibody ELISA cut-off ratios from venous serum samples versus dried blood sample from OneDraw device from second feasibility study

Suppl Fig 5: Validation study of blood sample collected by finger prick as dried blood Spot (BDS) versus venous serum sample.

Further Supplementary information

Instruction for use of the COVID-19 IgG ELISA Kit (Qualitative Assay for COVID-19 IgG) ODL150/10

Standard Operating Procedure for the COVID-19 IgG ELISA test of dried blood samples

Methods

The Drawbridge OneDraw device is licensed for use by health care professionals to collect samples for the measurement of HbA1c. This device has been FDA 510k approved (6) and CE marked.(7) To use the device in the Fenland COVID-19 study we applied for dispensation from the UK Medicines and Healthcare products Regulatory Agency, to change the CE licensed use in a limited way to allow the use of the device at home by a non-healthcare professional for blood sampling.

The OneDraw device is placed on the lateral upper arm and creates a small vacuum to stabilise the device on the skin, after which a needle pierces the skin and approximately 150µL of blood is collected onto two paper strips (8 x 22mm), which sit in a removable cartridge. The needle fully retracts into the device, which can then be safely disposed of in a sharps bin. The blood collection is complete when the two paper strips turn red in colour, which is seen through the visual indicator window (1-10 minutes). The device is removed by peeling away from the skin, the cartridge taken out of the device and inserted into a transport sleeve. It can then be safely sent in the postal service in a small package to the laboratory. Within the transport sleeve there is desiccant that enables the blood sample to dry further on the paper during this period. The resulting blood sample is very similar to a dried blood spot, with two paper strips saturated with capillary whole blood. Further information on the OneDraw device is given in the further supplementary information.

Feasibility study

The feasibility assessed if the OneDraw device could be used to obtain a standardised and adequate dried blood sample in the participant's home without the

help of a HCP from two body sites. We developed a revised protocol and modified the instructions for use at the upper arm site for use by a non-HCP with assistance from a household member and we developed instructions for use for a second body site on the thigh, for application for those who do not have assistance (Suppl. Fig. 1). For the Fenland COVID-19 study we also developed online instruction videos (https://www.mrc-epid.cam.ac.uk/research/studies/fenland-covid19/information-for-participants/information-for-all-participants/onedraw-videos/). Participants were recruited from staff members and local people living nearby, as travel restrictions were in place at the time of recruitment. Adults (>18 years) were recruited if they were able to speak and understand written and spoken English and follow written instructions. Participants were excluded if they were displaying symptoms of COVID-19 or had a confirmed COVID-19 positive antigen or PCR test in the previous 14 days.

Participants (n=41) were provided with two OneDraw device sample collection kits, instructions for use, a sharps bin for disposal of the devices and two return envelopes to send their blood samples for processing. They were asked to (1) use one of the devices on their upper arm with someone in their household to help them apply the device and (2) self-apply the second device on their thigh. If any participants lived alone, they were asked to self-apply both devices to their thigh to assess this self-administration option. Participants were asked to use the site-specific instructions provided to do the blood draws and to undertake the blood draws on 2 separate occasions either on the same day or two consecutive days. To test the stability of the dried blood spot (DBS) sample after a period of time in transit, participants sent the samples back in the postal service in two separate envelopes to

our testing lab using the envelopes provided. Participants were asked to submit feedback on the clarity of the instructions for use and to complete a perceived pain scale using a numeric rating scale marked 1-10, where 1 (low) to 10 (high) for each administration body site. (8)

A sub-group of 21 participants additionally came to the clinical facility for standardised blood collection by a trained research team member using three methods in a randomised order; (1) OneDraw device on the upper arm (2) finger-prick and (3) blood sample from venepuncture. Participants were asked which method they preferred and to complete the same perceived pain scale for each method. The stability of the samples collected remotely were compared to those collected in standardised conditions.

Validation study

Participants (n=120) were recruited from a population who had previously been tested for SARS-CoV-2 IgG antibodies and tested negative or positive, with and without previous definitive COVID-19 symptoms. Recruitment occurred in an approximate 2:1 ratio of participants who had previously tested positive and negative for antibodies (based on a range of assays) respectively to ensure a sufficient range of IgG antibody levels in the study sample. Recruitment criteria was the same as for the feasibility study.

We compared antibody test results from blood samples taken using (1) the OneDraw device from the upper arm (2) finger-prick and (3) blood sample from venepuncture, all undertaken at the clinical facility by a trained research team member.

Sample size for the validation study was based on estimating the correspondence of the COVID-19 IgG antibody status between venous samples and the dried blood spot samples taken by the OneDraw device across two independent samples of individuals (COVID-19 antibody negative and COVID-19 antibody positive participants). The study was not designed to investigate the accuracy of the test, but to assess the agreement in the categorisation of SARS-CoV-2 IgG antibody status between serum and OneDraw sample. Although it would be desirable to have a perfect alignment we wanted to make sure that there was an optimal sample number to obtain a Cohen's Kappa (κ) of $\kappa = 0.95$, requiring an observed agreement of $p_0 = 0.975$. (20) For an SE(κ) to be <0.05, we required an n=40 (SE = 0.049) based on pE = 0.5 (chance agreement).

Fenland COVID-19 study

The Fenland COVID-19 study involves participant of the Fenland cohort study, which was established in 2005, recruiting participants from GP registers across Cambridgeshire born between 1950 and 1975. The Fenland cohort study was designed to investigate the interaction between environmental and genetic factors in determining obesity, type 2 diabetes and related metabolic disorders. Participants were excluded if they had a known diagnosis of diabetes, a terminal illness with a prognosis of less than 1 year, psychotic illness, being pregnant or lactating or being unable to walk unaided. Between 2005-2015, 12,435 participants took part in phase 1 of the study and 7,795 participants took part in phase 2. At both phases, participants attended a clinical visit at one of the Cambridgeshire research sites where physiological measures and questionnaires were completed and blood

samples taken. Phase 2 clinical visits started in 2014 and were halted at the start of the UK COVID-19 pandemic in March 2020.

The primary objective of the Fenland COVID-19 study was to assess SARS-CoV-2 antibody status from the blood samples collected at 0, 3 and 6 months after initial recruitment in July 2020 in participants from an on-going population-based cohort study in Eastern England. Participants were recruited from the existing Fenland cohort study. Participants who had not died or withdrawn and had a valid telephone number or email address (n=11,469) were invited via email, text message or telephone call to take part in the Fenland COVID-19 study from July 2020. There were no exclusion criteria applied. Participants were asked to complete questionnaires on their health, diet, physical activity and body weight at regular intervals during the study to assess the impact of the COVID-19 pandemic and government restrictions on their health and behaviours (Suppl Fig. 2).
Participants consented into the study from 5th July 2020. Due to the timing of the UK rollout of SARS-CoV-2 vaccines in this age group, it was decided by the research project team to end the data collection period on the 30th April 2021 once all participants had reached the 6-month blood sample collection timepoint to measure SARS-CoV-2 IgG antibodies.

Socio-demographic and self-reported health characteristics

Information on occupation and education attainment level had been previously collected in the Fenland cohort study. Occupation was classified into three socio-economic categories; (1) low: routine manual and service, semi-routine and technical; (2) medium: middle or junior managers, clerical and intermediate (3) high: traditional professional, modern professional and senior managers. (21) Education was categorised on the basis of having a university degree (yes/no). The English

indices for multiple deprivation (IMD) was derived from the participants' postcode using the 2019 criteria based on 7 domains of deprivation. (22) Area deprivation was classified as whether the IMD score was within the lowest 30% most deprived areas nationally (yes/no). Participants completed questions on smoking habits (current smoker, ex-smoker and non-smoker) and self-reported health quality in the baseline questionnaire (poor, fair, good or excellent).

Laboratory analysis

Upon receipt of the blood samples (both OneDraw strips and dried blood spots from finger pricks) at the laboratory, they were checked for dryness and dry samples were stored in a zip lock bag at – 70 ^{0}C until analysis. Serum samples were stored in 1.5ml cryovials at – 70 ^{0}C until analysis.

We developed an extraction method for the capillary samples for enzyme-linked immunosorbent assay (ELISA) based antibody assay and validated this method for SARS-CoV-2 serology.(9)

The manufacturer developed and validated the assay for serum (or plasma). At the time of planning, the available MHRA approved serology assays were only for high through-put analysers. The nature of DBS sample required the use of an ELISA adaptable to samples extracted from DBS. Omega/Mologic provided data on the evaluation of their assay in diverse clinical settings in partnership with WHO centres internationally. We used the samples of the feasibility study in the sub-group who came to the clinical facility for standardised blood collection to assess if dried blood samples could be used for antibody status measurement using an ELISA method.

The samples were analysed for SARS-CoV-2 IgG antibodies using a commercial enzyme linked-immunosorbent assay (ELISA), which targets two viral antigens for

better sensitivity and specificity: NP (Nucleocapsid protein) and S2 (domain 2 spike protein) from SARS-CoV-2 (Omega diagnostics, UK). We followed a standard operating procedure (in the supplementary information) for extraction of the dried blood sample interfacing with the semi-quantitative Omega/Mologic COVID-19 IgG assay.

For the antibody status measurement, we extracted one dried blood strip (ca 75 µL) in 1.8 mL of phosphate buffered saline (PBS) overnight and the aqueous extract was then further diluted and analysed according to kit method. Repeat analysis of the same samples showed that 1-3 months storage at -70 ^{0}C or at room temperature (18 ^{0}C-25 ^{0}C) had no impact on the IgG measurement (Suppl Fig. 4a), which provided the assurance that samples collected could be batched up and analysed at regular intervals.

Each partially filled sample was graded (Suppl Fig. 3a) based on visual determination of the volume of blood on the strip. The gradings were used to enable the extraction of partially filled strips with a volume of PBS proportional to the amount of blood on the strip.

Data analysis

Wilcoxon signed rank test was used to assess within-person pain score values. Chi-square tests were used to assess differences in categorical variables between those who had a valid antibody result with those who did not complete a sample at each timepoint and ANOVA for continuous variables. The COVID-19 antibody test results from the dried blood spot samples collected by finger-prick and by the OneDraw device were compared with the venous test in all groups combined (n=120) by calculating Cohen's kappa coefficient, together with a 95% confidence interval.

Agreement between the dried blood spot cut-off ratio with the serum cut-off ratio was assessed with Pearson correlations and Bland Altman plots (expressed as constant and proportional).

Studies and ethics

Ethics approval for the feasibility study was granted by the University of Cambridge Human Biology Research Ethics Committee and for the validation study by the HRA and Health and Care Research Wales. Ethics approval for the Fenland COVID-19 study was granted by Southwest Cornwall and Plymouth Research Ethics committee. The Fenland Cohort participant panel was involved in the planning, conducting and reporting of the Fenland COVID-19 study. All participant provided informed consent using a secure online form.

Suppl Table 1: Pain scores and success rate of blood collection using the OneDraw device on the upper arm and thigh in feasibility study

	Successful blood collections, (n (%))	Pain score[1], median (Interquartile range)
Thigh (n=41)	37 (90%)	2 (1-2)
Arm (n=39)	36 (92%)	2 (1-2)

[1]: Pain scale on a score from 1-10 (1=lowest, 10 highest).

Suppl Table 2: Comparison of blood collection using OneDraw device with other blood draw methods: pain score and acceptability (n=21)

	**OneDraw**	**Venous**	**Finger-prick**
Pain score[1]	2 (1-2)	3 (2-4)	3 (2-4)
Preferred modality	76%	24%	0%

[1]: Pain scale on a score from 1-10 (1=lowest, 10 highest).

Suppl Table 3: Comparison of socio-demographic characteristics of Fenland COVID-19 study participants with valid antibody result with those who did not complete blood sample at each timepoint (0, 3 and 6 months)

	**Baseline**		**3-months**		**6-months**	
Returned sample	Yes<br>N=3,702	No<br>N=321	Yes<br>N=3,492	No<br>N=426	Yes<br>N=3,453	No<br>N=278
Age* Mean (SD)	59.0 (7.0)	58.3 (7.0)	59.1 (7.0)	57.1 (7.0) [A]	59.1 (7.0)	56.5 (7.0) [A]
Men N (%)	1614 (43.6)	144 (44.9)	1518 (43.5)	208 (48.8) [B]	1499 (43.4)	134 (48.2)
Smoking status N (%):						
Current smoker	133 (3.8)	7 (4.9)	118 (3.6)	19 (6.8) [B]	108(3.3)	26 (11.5) [A]
Ex-smoker	1,250 (35.8)	38 (26.4)	1,191 (35.8)	87 (31.1)	1,162 (35.3)	92 (40.5)
Non-smoker	2,113 (60.4)	99 (68.8)	2,015 (60.6)	174 (62.1)	2,020 (61.4)	109 (48.0)
High self-rated health N (%)	3,425 (99)	134 (97.8)	3,251 (98.9)	274 (98.9)	3,223 (99.0)	218 (97.3) [B]
SES category n(%):						
Traditional and modern professional and higher managerial	2320 (64.8)	201 (65.3)	2199 (65.2)	264 (63.8) [B]	2172 (65.1)	170 (63.2)
Lower managerial and intermediate occupations	680 (19.0)	56 (18.2)	646 (19.1)	64 (15.5)	634 (19.0)	44 (16.4)
Traditional and modern professional and higher managerial	580 (16.2)	51 (16.6)	530 (15.7)	86 (20.8)	531 (15.9)	55 (20.5)
University degree	1668 (45.1)	149 (46.6)	1595 (45.7)	176 (41.3)	1570 (45.5)	115 (41.4)
Residence in 30% most deprived areas	447 (12.2)	39 (12.2)	413 (11.9)	61 (14.4)	406 (11.8)	41 (14.8)

Data available at baseline- smoking status n=3,640; self-rated health n=3,598; socio-economic Status (SES) class n=3606; University degree 3727; IMD deprivation classification n=3,706;

[A] $p<0.001$ [B] $p<0.05$

Suppl Table 4: Number of replacement OneDraw devices sent

Number of Replacements	Participants at Baseline	Participants at 3-months	Participants at 6-months
	N	N	N
1	385	463	261
2	45	40	8
3	1	5	2

Suppl. Fig 1a: Instructions for use for blood collection form upper arm using OneDraw device.

MRC | Epidemiology Unit

UNIVERSITY OF CAMBRIDGE

OneDraw Blood Collection Device

Upper arm application – Instructions for use

Please read these instructions carefully and ***all the way through*** before you begin taking a sample.

Introduction

The OneDraw device applies a vacuum to the skin and two small lancets that make an incision on the surface of the skin. The device will collect approximately 150 microlitres (about 3 drops) of blood. This blood is collected on strips of filter paper that can be sent back in the post for testing.

These instructions are designed for administration of the device on your upper arm, with the help of someone in your household. If you are self-administering on your thigh, please use the separate thigh instructions.

Please try to take your sample as soon as you can upon receipt of the device, and post your sample back to us the next day (ideally **between Sunday and Thursday**).

We advise you to set aside 20 minutes to complete the entire process, including the time to read these instructions. You will need to **sit still for the blood draw portion of this time.** You should have a clock or watch so that you know if the sample is taking too long (longer than five minutes for blood to appear in the upper part of the chamber window).

***Videos on how to use the device can be found on our website* www.mrc-epid.cam.ac.uk/fenland-covid19/**

Pack contents

The pack you have received should contain the following items. If anything is missing, please contact us as soon as possible so we can arrange a replacement.

These instructions for use of device. *Upper arm instructions strongly recommended for use if someone can assist you.*	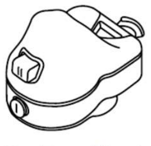 OneDraw blood collection device	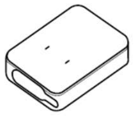 Transport sleeve	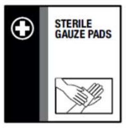 Gauze	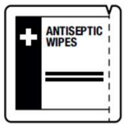 Antiseptic wipes
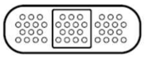 Plaster (Do not use if you have a latex allergy)	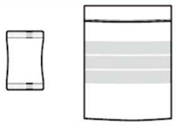 Arm/leg labelled plastic bag with gel dessicant and transport sleeve to go inside.	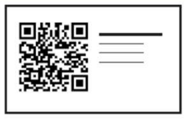 QR barcode labels x 3 (One for transport sleeve, one for plastic bag, one spare)	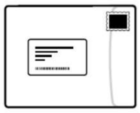 Freepost jiffy bag for sample return	

You will also need:

- Extra person to assist you (for upper arm application of device only)
- Chair to sit quietly for blood draw
- Clock or watch
- Latex-free plaster (if you have a latex allergy)
- Sharps bin (provided in your first kit)

1 OPEN PACKAGE

Remove package contents. Remove the foil lid from plastic tray by peeling from the corner.

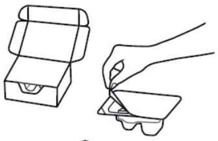

2 PREPARE TRANSPORT SLEEVE

Apply a QR barcode label onto the flat side of the sample transport sleeve covering the patient name and DOB sticker. **DO NOT write your name or DOB on the transport sleeve.** Remove the foil completely from the end of the sample transport sleeve.

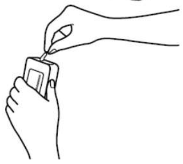

3 SELECT AND CLEAN COLLECTION LOCATION

Select a collection location on the outside of the upper arm, approximately 2-4 inches (5-10 centimetres) below the top of the shoulder.

Clean the collection area with an antiseptic wipe. Allow to air dry.

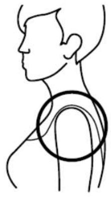

4 REMOVE ADHESIVE LINER

Remove the adhesive protection liner from the bottom of the device.

Retain the adhesive liner for Step 11.

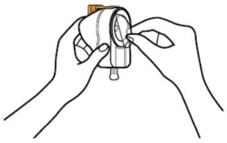

5 APPLY DEVICE TO COLLECTION LOCATION

Apply the device to the skin at the selected location on your upper arm. The vacuum activation button must be facing upward. (The device should be oriented vertically regardless of your orientation.)

Ensure the device is flush, you can see the visual indicator window clearly, and the entire bottom surface is in contact with the skin.

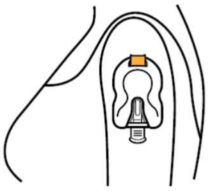

6 REMOVE THE YELLOW BUTTON LOCK

Remove the button lock from the device by pulling it straight away from the device and the arm.

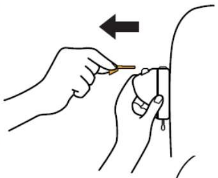

7 ACTIVATE THE VACUUM (BUTTON “I”)

While holding the device in place, **firmly** press Button ‘I’ (the button closest to the skin) to activate the vacuum. **This button may be stiff and difficult to push.** When completely depressed, the button will become flush with the surrounding surface.

Wait 30 secs before moving onto the next step to allow the device to fully suction to your skin.

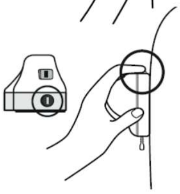

8 ACTIVATE THE LANCET (BUTTON “II”)

While again holding the device in place, **firmly** press Button ‘II’ (the button furthest from the skin) to activate the lancet.**This button may be stiff and difficult to push.**

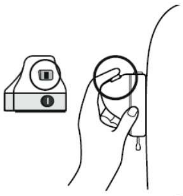

9 WAIT FOR BLOOD COLLECTION TO COMPLETE

Wait for the blood collection to complete (approximately 1-10 minutes) while observing the visual indicator window. Adhesive will hold the device in place.

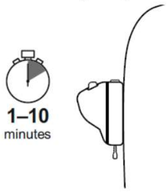

10 COMPLETE BLOOD COLLECTION

The blood collection is complete when the blood collection matrix that can be seen through the visual indicator window turns completely **RED** in colour.

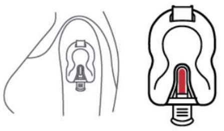

11 PROMPTLY REMOVE DEVICE

Once the visual indicator window has turned completely red, promptly remove the device by gently peeling away from the skin to release the adhesive and vacuum seal.

Reapply the release liner (saved from step 4) and place the device on a level surface, adhesive side down.

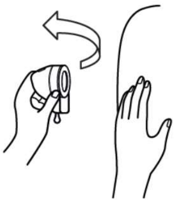

Please make sure to remove the device from the skin promptly upon completion of the blood collection. If the cartridge overfills, the rear oval window of the device may appear completely red or pooled blood may be visible through the oval window. If this occurs, remove the cartridge slowly to avoid splash or spill of excess blood.

12 CLEAN BLOOD COLLECTION SITE

Wipe remaining blood from the collection site using a clean gauze pad, pressing firmly to stop blood flow. Place an adhesive bandage on the collection area. **If you have a latex allergy, please do not use the plaster provided.**

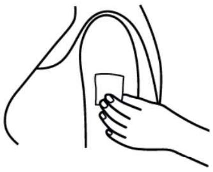

13 REMOVE CARTRIDGE

Remove the cartridge from the device by pulling on the rear tab and sliding it outward from the slot. **Always remove the cartridge slowly to avoid splash/spill of excess blood.** The cartridge may be difficult to remove from the device, this is normal.

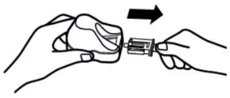

In some cases, only one side of the cartridge appears red upon completion of the blood collection, indicating that only one of the two filter paper matrix strips has been saturated. One fully saturated matrix is sufficient to ship the sample.

14 PREPARE SAMPLE FOR POSTING

Insert the cartridge into the transport sleeve by sliding it into the slot completely. The cartridge may be difficult to slide into the transport sleeve, this is normal. Use your thumbs on either side of the handle to help push the cartridge into the sleeve. You may hear a “click” sound when the cartridge is fully inserted.

Place the transport sleeve and gel dessicant into the clear plastic bag and seal it. Apply a QR barcode label on the outside of the bag.

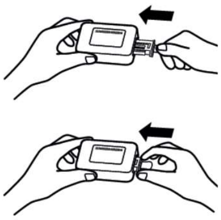

15 POSTING THE DEVICE

Place the labelled plastic bag containing your sample and dessicant into the pre-paid envelope and post to the laboratory for testing. Please do this on the same day as the sample was taken. Please do not include any notes with the sample.

If you need to let the team know about something, please get in contact via email. The return sample package will fit into a post box, you do not need to go into a post office to send it.

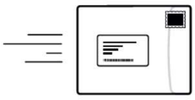

16 DEVICE DISPOSAL

Dispose of the device in the biohazardous waste sharps container. Please see further instructions overleaf. The rest of the items can be disposed of in domestic waste.

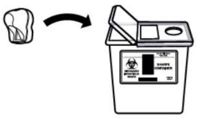

OneDraw Blood Collection Device

Thigh application – Instructions for use

Please read these instructions carefully and ***all the way through*** before you begin taking a sample.

Introduction

The OneDraw device applies a vacuum to the skin and two small lancets that make an incision on the surface of the skin. The device will collect approximately 150 microlitres (about 3 drops) of blood. This blood is collected on strips of filter paper that can be sent back in the post for testing.

These instructions are designed for self-administration of the device on your thigh. If you have someone in your household who can help you administer the device, **we would prefer you to use the upper arm location for the blood collection**. See separate instructions for the upper arm.

Please try to take your sample as soon as you can upon receipt of the device, and post your sample back to us the next day (ideally **between Sunday and Thursday**).

We advise you to set aside 20 minutes to complete the entire process, including the time to read these instructions. You will need to **sit still for the blood draw portion of this time.** You should have a clock or watch so that you know if the sample is taking too long (longer than five minutes for blood to appear in the upper part of the chamber window).

Videos on how to use the device can be found on our website **www.mrc-epid.cam.ac.uk/fenland-covid19/**

Pack contents

The pack you have received should contain the following items. If anything is missing, please contact us as soon as possible so we can arrange a replacement.

These instructions for use of device.<br>*Upper arm instructions strongly recommended for use if someone can assist you.*	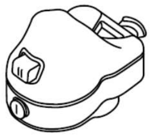<br>OneDraw blood collection device	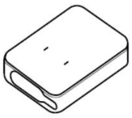<br>Transport sleeve	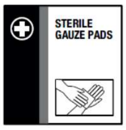<br>Gauze	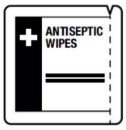<br>Antiseptic wipes
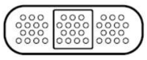<br>Plaster<br>(Do not use if you have a latex allergy)	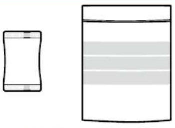<br>Arm/leg labelled plastic bag with gel dessicant and transport sleeve to go inside.	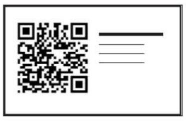<br>QR barcode labels x 3<br>(One for transport sleeve, one for plastic bag, one spare)	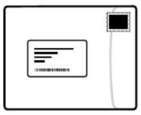<br>Freepost jiffy bag for sample return	

You will also need:

- Chair to sit quietly for blood draw
- Clock or watch
- Latex-free plaster (if you have a latex allergy)
- Sharps bin (provided in your first kit)

1 OPEN PACKAGE

Remove package contents.Remove the foil lid from the plastic tray by peeling from the corner.

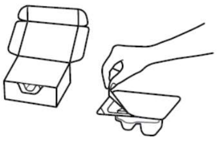

2 PREPARE TRANSPORT SLEEVE

Apply a QR barcode label onto the flat side of the sample transport sleeve covering the patient name and DOB sticker. **DO NOT write your name or DOB on the transport sleeve.** Remove the foil completely from the end of the sample transport sleeve.

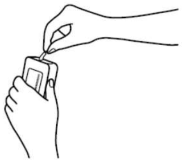

3 SELECT AND CLEAN COLLECTION LOCATION

Select a collection location on the lower part of your thigh, 6-7 inches (15-18 centimetres) from your knee. To take the sample please sit upright in a chair with your knee bent and your leg raised. Clean the collection area with an antiseptic wipe. Allow to air dry.

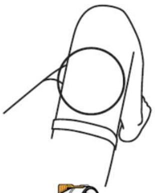

4 REMOVE ADHESIVE LINER

Remove the adhesive protection liner from the bottom of the device.
Retain the adhesive liner for Step 11.

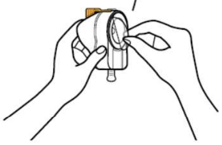

5 APPLY DEVICE TO COLLECTION LOCATION

Sit in a chair as pictured. Apply the device to the skin at the selected location on your thigh. The vacuum activation button must be facing upward. (The device should be oriented vertically regardless of your orientation.)

Ensure the device is flush, you can see the visual indicator window clearly, and the entire bottom surface is in contact with the skin.

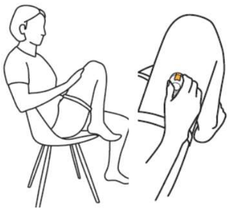

6 REMOVE THE YELLOW BUTTON LOCK

Remove the button lock from the device by pulling it straight away from the device and your leg.

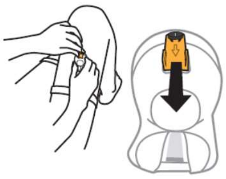

7 ACTIVATE THE VACUUM (BUTTON “I”)

While holding the device in place, **firmly** press Button ‘I’ (the button closest to the skin) to activate the vacuum. **This button may be stiff and difficult to push.** When completely depressed, the button will become flush with the surrounding surface.

Wait 30 secs before moving onto the next step to allow the device to fully suction to your skin.

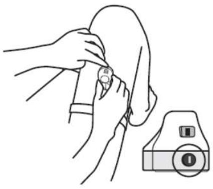

8 ACTIVATE THE LANCET (BUTTON “II”)

While again holding the device in place, **firmly** press Button ‘II’ (the button furthest from the skin) to activate the lancet.**This button may be stiff and difficult to push.**

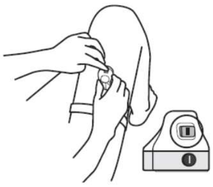

9 WAIT FOR BLOOD COLLECTION TO COMPLETE

Wait for the blood collection to complete (approximately 1-10 minutes) while observing the visual indicator window. Adhesive will hold the device in place.

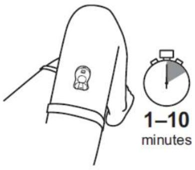

10 COMPLETE BLOOD COLLECTION

The blood collection is complete when the blood collection matrix that can be seen through the visual indicator window turns completely **RED** in colour.

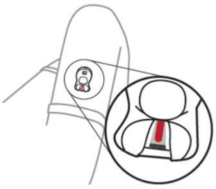

11 PROMPTLY REMOVE DEVICE

Once the visual indicator window has turned completely red, promptly remove the device by gently peeling away from the skin to release the adhesive and vacuum seal. Reapply the release liner (saved from step 4) and place the device on a level surface, adhesive side down.

Please make sure to remove the device from the skin promptly upon completion of the blood collection. If the cartridge overfills, the rear oval window of the device may appear completely red or pooled blood may be visible through the oval window. If this occurs, remove the cartridge slowly to avoid splash or spill of excess blood.

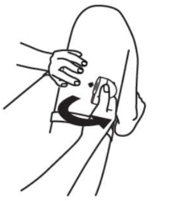

12 CLEAN BLOOD COLLECTION SITE

Wipe any remaining blood from the collection site using a clean gauze pad, pressing firmly to stop blood flow. Place an adhesive bandage on the collection area. **If you have a latex allergy, please do not use the plaster provided.**

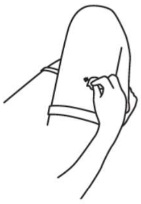

13 REMOVE CARTRIDGE

Remove the cartridge from the device by pulling on the rear tab and sliding it outward from the slot. **Always remove the cartridge slowly to avoid splash/spill of excess blood.** The cartridge may be difficult to remove from the device, this is normal.

In some cases, only one side of the cartridge appears red upon completion of the blood collection, indicating that only one of the two filter paper matrix strips has been saturated. One fully saturated matrix is sufficient to return the sample.

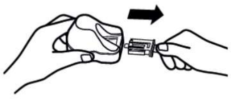

14 PREPARE SAMPLE FOR POSTING

Insert the cartridge into the transport sleeve by sliding it into the slot completely. The cartridge may be difficult to slide into the transport sleeve, this is normal. Use your thumbs on either side of the handle to help push the cartridge into the sleeve. You may hear a “click” sound when the cartridge is fully inserted.

Place the transport sleeve and gel dessicant into the clear plastic bag and seal it. Apply a QR barcode label on the outside of the bag.

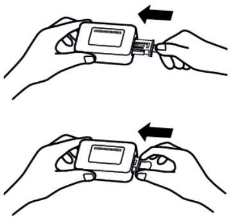

15 POSTING THE DEVICE

Place the labelled plastic bag containing your sample and dessicant into the pre-paid envelope and post to the laboratory for testing. Please do this on the same day as the sample was taken. Please do not include any notes with the sample.

If you need to let the team know about something, please get in contact via email. The return sample package will fit into a post box, you do not need to go into a post office to send it.

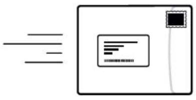

16 DEVICE DISPOSAL

Dispose of the device in the biohazardous waste sharps container. Please see further instructions overleaf.The rest of the items can be disposed of in domestic waste.

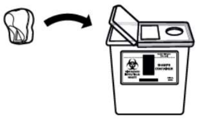

Suppl Fig 2. Fenland-COVID study design. At three time points participants were asked to provide a blood samples collected at home using the OneDraw device. At the same time participants were asked to complete a on-line Diet and Physical Activity questionnaire and every month COVID-19 questionnaire and body weight. For all participants COVID-19 test results collected by Public Health England were retrieved for the period of the study (July 2020-May 2021).

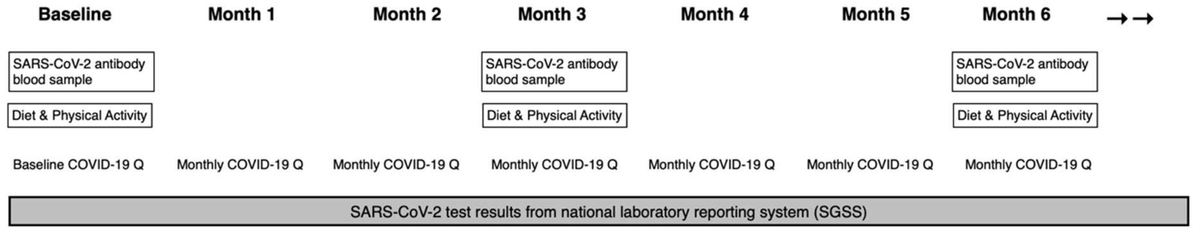

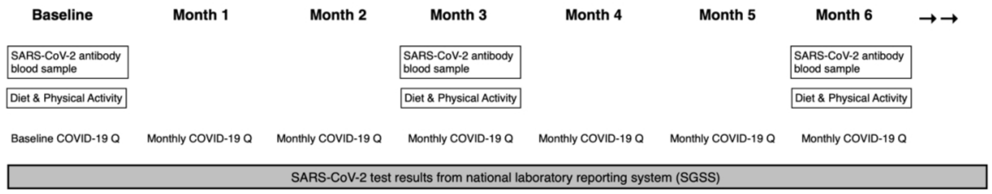

Suppl Fig 2. Fenland-COVID study design. At three time points participants were asked to provide a blood samples collected at home using the OneDraw device. At the same time participants were asked to complete a on-line Diet and Physical Activity questionnaire and every month COVID-19 questionnaire and body weight. For all participants COVID-19 test results collected by Public Health England were retrieved for the period of the study (July 2020-May 2021).

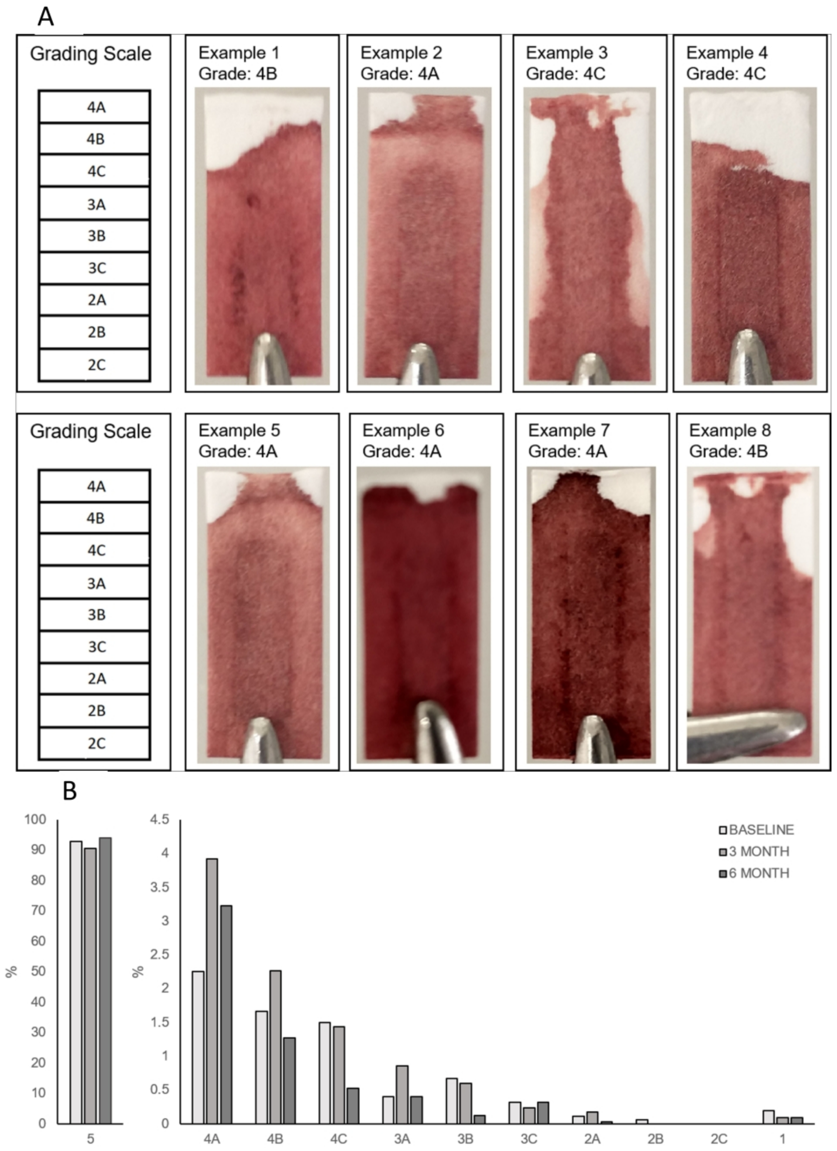

Suppl Fig 3: Partially filled strips; A) examples of partially filled strips and their grading; B) percentage of samples at Baseline (n=3735), 3 Months (n=3497) and 6 Months (n=3470) that were partially filled, 5=full, 1=empty, the other levels are on grading scale.

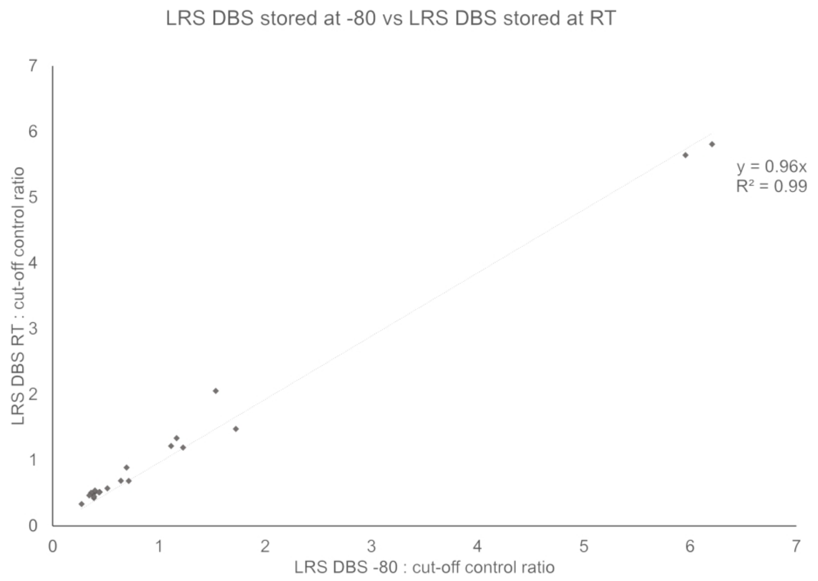

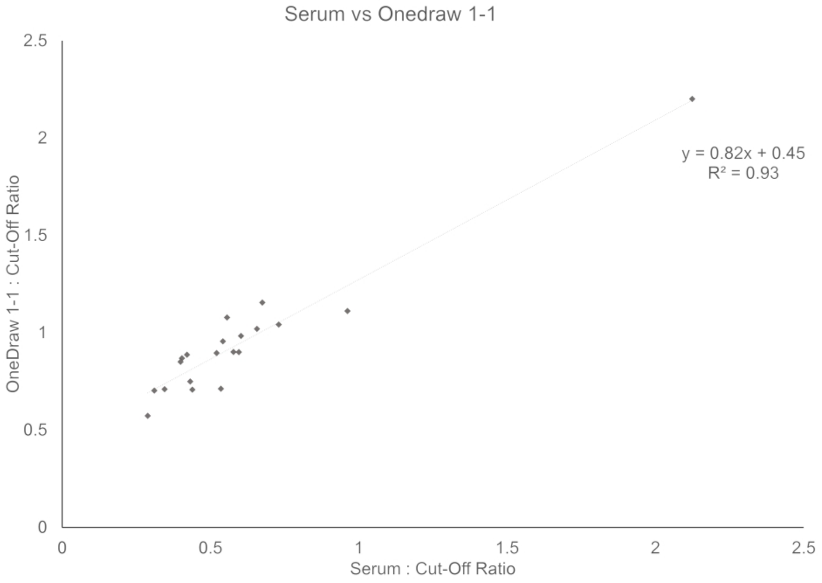

Caption : Suppl fig 4a: Correlation between SARS-CoV-2 antibody ELISA cut-off ratios from EDTA plasma samples spotted on dried blood spot cards, stored at room temperature (RT) versus dried samples stored at -80 ^{0}C for 1-3 months. Samples provided by the NIHR Bioresouce Cambridge.

Suppl fig 4b: Correlation between SARS-CoV-2 antibody ELISA cut-off ratios from venous serum samples versus dried blood sample from OneDraw device from second feasibility study (n=21).

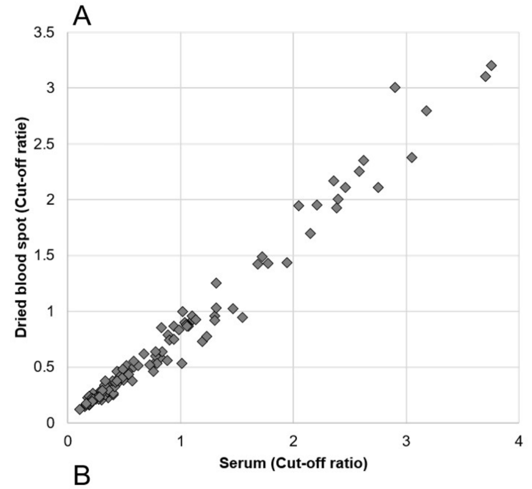

B

DBS	serum pos	serum neg	serum BL
pos	20	0	0
neg	2	75	7
BL	7	0	9

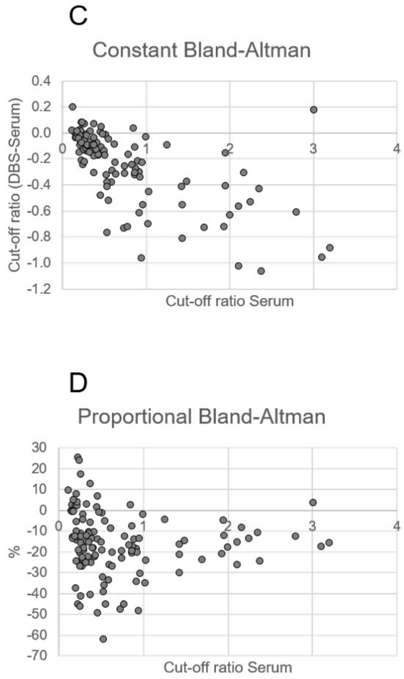

Suppl Fig 5. Validation study of blood sample collected by finger prick as dried blood Spot (BDS) versus venous serum sample. A) Correlation between SARS-CoV-2 IgG antibody ELISA cut-off ratios from venous serum samples versus dried blood spot (R2=0.98; n=120); B) confusion matrix of the classification of the results (n=120, pos=positive (cut-off ratio > 1.1, neg=negative (cut-off ratio <0.8, BL=borderline (cut-off ratio <1.1 & >0.8); C) Constant Bland-Atlman plot and D) proportional Bland-Atlman plot showing the deviation of the SARS-CoV-2 IgG antibody ELISA results for from dried blood spot samples (n=120).

Omega DIAGNOSTICS LTD

en

COVID-19 IgG ELISA Kit

REF ODL150/10

Qualitative Assay for COVID-19 IgG
Store at 2-8°C. DO NOT FREEZE.
For professional use only.

IVD CE

Σ 960

INTENDED USE

The test is a qualitative ELISA kit for the detection of IgG antibodies to SARS-CoV-2 in human venous serum, venous plasma (EDTA, lithium heparin, sodium citrate) or capillary plasma (lithium heparin). Capillary plasma is plasma obtained from capillary whole blood. The COVID-19 IgG test is an aid to identification of recent or prior infection of SARS-CoV-2 in symptomatic and asymptomatic individuals who may have gained an adaptive immune response. Test results should be considered in conjunction with other test results/clinical information and should not be used solely for diagnosis nor to exclude acute infection.

Note. Detection of antibodies may indicate immunity or suspected attenuation to subsequent re-infection. The length of time SARS-CoV-2 antibodies remain in the body post infection is unknown.

The COVID-19 IgG ELISA assay should not be used to diagnose acute SARS-CoV-2 infection, if acute infection is suspected, direct testing for SARS-CoV-2 is necessary.

INTRODUCTION

Severe Acute Respiratory Syndrome CoronaVirus 2 (SARS-CoV-2) is the viral strain that causes coronavirus disease 2019 (COVID-19). Following infection, the immune system may generate antibodies to fight the infection. The test can detect immunoglobulin G (IgG) antibodies to SARS-CoV-2. Levels of IgG antibodies develop and decline at different rates within different individuals. The immunological response to viral infection can take several weeks and evidence suggests that antibodies to SARS-CoV-2 may take several days to appear. Serological testing of an individual before this window period may result in a misleading negative result.

Detection of IgG antibodies may indicate immunity or attenuation to subsequent re-infection for the individual and for seroprevalence studies to provide a measure of how many people have been exposed to SARS-CoV-2 (in the absence of other, e.g. antigen, test results).

PRINCIPLE OF THE TEST

The test is a serological, plate-based, enzyme linked immunosorbent assay (ELISA), for detecting and quantifying SARS-CoV-2 IgG in human venous serum, venous plasma (EDTA, lithium heparin, sodium citrate) or capillary plasma (lithium heparin). Capillary plasma is plasma obtained from capillary whole blood.

Diluted serum/plasma samples are incubated with SARS-CoV-2 antigens immobilised on microtitre wells. After washing away unbound serum/plasma components, anti-human IgG conjugated to horseradish peroxidase is added to the wells, and this binds to surface-bound antibodies in the second incubation. Unbound conjugate is removed by washing, and a solution containing 3,3',5,5'-tetramethylbenzidine (TMB) and enzyme substrate is added to indicate antibody binding. Addition of Stop Solution terminates the reaction and provides the appropriate pH for colour development. The optical densities of the cut-off control, positive control and samples are measured using a microplate reader at 450nm.

CONTENTS OF THE COVID-19 IgG ELISA KIT

Materials provided:

- **MTP** - 10 96-well microtitre plate: pre-coated with purified SARS-CoV-2 antigens (NP and S2 antigens), in a foil bag with desiccant
- **DIL** - 1 Sample Diluent: 150mM Tris-buffered saline, pH 7.2 with antimicrobial agent, 120mL, (blue), concentrate (15×)
- **WB** - 6 Wash Buffer: 100mM Tris-buffered saline with detergent, pH 7.2, 170mL, concentrate (10×)
- **CONJ** - 1 Conjugate: Anti-human IgG conjugated to horseradish peroxidase in protein stabilising solution and antimicrobial agent, 120mL, ready to use
- **SUBS** - 1 Substrate: aqueous solution of TMB and hydrogen peroxide, 120mL, ready to use
- **STOP** - 1 Stop Solution: 0.25M sulphuric acid, 120mL, ready to use
- **CONTROL CO** - 2 Cut-off Control: of 10mM Tris-buffered saline containing purified human IgG antibodies, 3mL, ready to use
- **CONTROL +** - 2 Positive Control: of 10mM Tris-buffered saline containing purified human IgG antibodies, 3mL, ready to use
- Instructions for use

Materials required, but not provided:

- Test tubes for dilution
- Graduated cylinder for preparing wash buffer and sample diluent
- Precision pipettes and disposable tips to deliver 5µL, 100µL, and 1mL
- Enzyme Immunoassay (EIA) microplate washer or multi-channel pipette or wash bottle
- Distilled or de-ionised water
- Absorbent paper
- EIA microplate reader with 450nm and optional 620nm reference filter. Alternatively, a suitable, self-validated automated system may be used.

Instrumentation, whether manual or automated, should meet the following criteria: pipettes with better than 3% imprecision with no carry over between pipetting steps; microplate washers should remove 99% of fluid; automated machines should minimise time between washing and adding the next reagent.

KIT STORAGE

On arrival, store the kit at 2-8°C. Do not use kits beyond their expiry date. Do not freeze any kit component.

QUALITY CONTROL

The expected OD values and the acceptance ranges for the Cut-off Control and Positive Control are given on the Quality Control Certificate included in the kit. The Positive Control is intended to monitor for substantial reagent failure. Any well positive by spectrophotometer but without visible colour should be cleaned on the underside and re-read. If OD values below zero are observed, the wavelengths used should be verified, the reader re-blanked to air and the measurements repeated.

SAMPLE COLLECTION AND TEST PROCEDURE

Human venous serum, venous plasma (EDTA, lithium heparin, sodium citrate) or capillary plasma (lithium heparin) samples can be used. Capillary plasma is plasma obtained from capillary whole blood. Serum and plasma samples should be stored at -20°C for long-term storage. Frozen samples must be mixed well after thawing and prior to testing. Repeated freezing and thawing can affect results. Capillary blood collected into lithium heparin blood collection tubes can be stored for up to 7 days at 25°C prior to centrifugation and plasma collection. Addition of preservatives to the serum sample may adversely affect the results. Microbially contaminated, heat-treated or specimens containing particulate matter should not be used. Do not use grossly haemolysed, icteric or lipaemic samples. Do not freeze whole blood samples.

Preparing for the test

1. Allow the test kit to come to a temperature between 16-30°C before use.
2. Dilute the Sample Diluent **(Reagent 1 = DIL)** 1 in 15 in distilled water to make sufficient buffer for the assay run e.g. add 10mL Sample Diluent concentrate to 140mL water.
3. Dilute the Wash Buffer **(Reagent 2 = WB)** 1 in 10 in distilled water to make sufficient buffer for the assay run e.g. add 100mL Wash Buffer concentrate to 900mL water.
4. Ensure that the microtitre plate is correctly orientated.

Test procedure

5. Dilute samples 1 in 201 in diluted Sample Diluent (e.g. 5µL serum added to 1mL diluted Sample Diluent).
6. Dispense 100µL of the Sample Diluent, Cut-off Control, Positive Control, and the diluted samples into appropriate wells. It is recommended that all controls and samples are run in duplicate.
7. Incubate for **30 minutes** at a temperature between 16-30°C.
8. After 30 minutes, decant or aspirate the well contents and wash the wells 3 times with diluted Wash Buffer using automated washing or the manual wash procedure (see below). Careful washing is the key to good results. Do not allow the wells to dry out.
9. Dispense 100µL of Conjugate (**Reagent 3 = CONJ**) into each well. Incubate the wells for **30 minutes** at a temperature between 16-30°C.
10. After 30 minutes, discard the well contents and carefully wash the wells 4 times with diluted Wash Buffer. Ensure that the wells are empty but do not allow to dry out.
11. Dispense 100µL of TMB Substrate (**Reagent 4 = SUBS**) into each well. Incubate the plate for **10 minutes** at temperature between 16-30°C.
12. Add 100µL of Stop Solution (**Reagent 5 = STOP**) to each well. To allow equal reaction times, the Stop Solution should be added to the wells in the same order as the TMB Substrate.
13. Read the OD of each well at 450nm in a microplate reader within **10 minutes**. A 620nm filter may be used as a reference wavelength.

Manual Wash Procedure

Empty the wells by inversion. Using a multi-channel pipette or wash bottle, fill the wells with diluted Wash Buffer (this is equivalent to approximately 300µL per well). Empty by inversion and blot the wells on absorbent paper. Ensure that the wells are empty but do not allow the wells to dry out. Insufficient washing may cause high background signal.

INTERPRETATION OF RESULTS

14. Results are expressed by calculating a ratio using the following formula:

Sample OD / (Cut-off Control OD * 1.6) = Sample Ratio

Interpretation of sample ratios is as follows:

Ratio	Interpretation
< 0.8	**Negative**, i.e. the subject's sample does not contain IgG antibodies to SARS-CoV-2
≥ 1.1	**Positive**, i.e. the subject´s sample contain IgG antibodies to SARS-CoV-2
≥ 0.8 to < 1.1	**Indeterminate**, if an indeterminate result is obtained, the test should be repeated using a new sample.

LIMITATIONS

- For use with human venous serum, venous plasma (EDTA, lithium heparin, sodium citrate) or capillary plasma (lithium heparin) samples only. Capillary plasma is plasma obtained from capillary whole blood.
- For reliable results, please follow the instructions carefully.
- Test results should be used in conjunction with other clinical and patient information.

WARNINGS

- Read the instructions carefully before performing the test. Failure to follow the instructions may lead to inaccurate test results.
- Use of any other components except those supplied with the kit will invalidate the results.
- Do not use the kit beyond the expiry date.
- Do not use if any kit components are damaged.
- Do not use if the product has been exposed to excessive heat or humidity.
- Do not use grossly haemolysed, icteric or lipaemic samples. Do not freeze whole blood samples.

SAFETY AND HANDLING PRECAUTIONS

- **Safety Precautions**
  - i. Handle all samples as potentially infectious.
  - ii. Wear gloves and protective clothing while handling samples and running the test.
  - iii. Do not smoke, eat, or drink while handling samples or performing the test procedure.
  - iv. Apply standard biosafety precautions for handling and disposal of potentially infective material as per local legislation. Dispose of all packaging in a general waste bin.
  - v. Avoid splashing and aerosol formation.
  - vi. Clean up spills thoroughly using an appropriate disinfectant.
- **Handling Precautions**
  - i. Do not use kit components beyond the expiry date printed on the label. Always check expiry date prior to testing.
  - ii. Ensure a new disposable pipette tip is used for each sample and disposed of following standard biosafety precautions.
  - iii. Sample Diluent concentrate (DIL) contains <0.1% sodium azide as a preservative which may be hazardous to health if ingested. Sodium azide may react with lead and copper plumbing to form highly explosive salts. On disposal, flush with large quantities of water.

PERFORMANCE CHARACTERISTICS

Performance Evaluation Data

All performance evaluations were collected by hospital clinic sites within the UK.

A total of 644 positive samples (laboratory confirmed SARS-CoV-2 by RT-PCR, ranging from day 0 to day > 60) and 642 negative samples (from pre-COVID-19 outbreak) were used, with the following results:

	%	95% Confidence Intervals
**Sensitivity** (total SARS-CoV-2 positive samples ranging from day 0 - >60 post laboratory diagnosis with RT-PCR)	91.1% (587/644)	88.68% - 93.23%
**Specificity** (total SARS-CoV-2 negative samples from pre-COVID-19 outbreak)	98.6% (633/642)	97.36% - 99.36%

Sensitivity stratified by days post swab for RT-PCR

Days post laboratory diagnosis with RT-PCR	Sensitivity (%)	95% Confidence Intervals
0-6	82.4% (28/34)	65.47% - 93.24%
7-13	91.5% (75/82)	83.20% - 96.50%
14-20	94.3% (83/88)	87.24% - 98.13%
21-27	96.0% (48/50)	86.29% - 99.51%
21-60	92.5% (74/80)	84.39% - 97.20%
> 21	91.4% (74/81)	83.00% - 96.45%

Sensitivity stratified by onset of symptoms

Days post symptom onset	Sensitivity (%)	95% Confidence Intervals
0-6	87.5% (7/8)	47.35% - 99.68%
7-13	87.9% (29/33)	71.80% - 96.60%
14-20	94.3% (66/70)	86.01% - 98.42%
21-27	93.7% (59/63)	84.53% - 98.24%
21-60	91.8% (90/98)	84.55% - 96.41%
> 21	92.0% (92/100)	84.84% - 96.48%

Cross Reactivity

Cross reactivity: SARS-CoV-2 negative samples that tested positive for a range of other viruses and disease states.

Source	Organism/condition	Negative agreement (%)
Resp Virus PCR	Adenovirus	100% (8/8)
Resp Virus PCR	Bocavirus	100% (2/2)
Serology	*Bordetella pertussis*	100% (22/22)
Resp Virus PCR	Coronavirus 229	94.4% (17/18)
Resp Virus PCR	Coronavirus 43	100% (12/12)
Resp Virus PCR	Coronavirus 63	100% (5/5)
Serology	Dengue virus	100% (16/16)
Resp Virus PCR	*Enterovirus*	100% (7/7)
Serology	Epstein-Barr Virus	100% (38/38)
Culture	*Haemophilus influenzae*	100% (17/17)
Resp Virus PCR	Hong Kong Uni coronavirus	100% (4/4)
Resp Virus PCR	*Influenza A*	94.1% (16/17)
Resp Virus PCR	*Influenza B*	100% (2/2)
Resp Virus PCR	Influenza H1N1	100% (12/12)
Urinary antigen	*Legionella*	100% (4/4)
TB culture	*Mycobacterium tuberculosis*	83.3% (10/12)
Malaria blood film	Malaria	100% (8/8)
Resp Virus PCR	Metapneumovirus	100% (9/9)
Resp Virus PCR	*Mycoplasma*	100% (2/2)
Resp Virus PCR	Parainfluenza 1	100% (3/3)
Resp Virus PCR	Parainfluenza 2	100% (6/6)
Resp Virus PCR	Parainfluenza 3	100% (8/8)
Resp Virus PCR	Parainfluenza 4	100% (4/4)
Resp Virus PCR	Parechovirus	100% (2/2)
PCR	*Pneumocystis jirovecii*	100% (6/6)
Resp Virus PCR	Respiratory Syncytial Virus	100% (12/12)
Serology	Rheumatoid factor	100% (27/27)
Resp Virus PCR	Rhinovirus	100% (12/12)
Serology	Serum save	100% (14/14)
Serology	Systemic Lupus Erythematosus	94.1% (16/17)

Source	Organism/condition	Negative agreement (%)
Culture	*Streptococcus pneumoniae*	100% (15/15)
Culture	*Strep. pyogenes* (Beta-haemolytic Strep group A)	100% (13/13)
	**TOTAL**	**98.6% (349/354)**

Interfering Substances

No interference in the performance of COVID-19 IgG kit was evident when SARS-CoV-2 positive and negative samples were spiked with the following interferents: Haemolysate up to 1mg/mL, Bilirubin (conjugated) up to 0.15mg/mL and Rheumatoid Factor up to 10IU/mL.

SYMBOL LEGEND

The following symbols have been used within the labelling of this product.

CE	This product fulfils the requirements of Directive 98/79/EC on in vitro diagnostic medical devices.	IVD	*In vitro* diagnostic medical device
			Keep away from sunlight
LOT	Batch code		Manufacturer
REF	Catalogue number		Temperature limit
	Consult instructions for use		Use-by date
Σ	Contains sufficient for "n" tests		

BIBLIOGRAPHY

1. *World Health Organisation* [online] 08 Apr 2020 (accessed: 24 April 2020): https://www.who.int/emergencies/diseases/novel-coronavirus-2019/technical-guidance/laboratory-guidance
2. Wölfel, R., Corman, V.M., Guggemos, W. *et al.* Virological assessment of hospitalized patients with COVID-2019. Nature (2020), viewed 21 May 2020, https://doi.org/10.1038/s41586-020-2196-x

263-ODL150/10 V6 FEBRUARY 2021

Genesis Diagnostics Ltd
Eden Research Park,
Henry Crabb Road,
Littleport,
Cambridgeshire
CB6 1SE
United Kingdom
+44 (0)1353 863220
support@elisa.co.uk
odl@omegadiagnostics.co.uk

Electronic instructions for use are available to view, download and print at:
www.omegadiagnostics.com

AN ISO 9001 AND ISO 13485 CERTIFIED COMPANY

Genesis Diagnostics Ltd is a subsidiary of Omega Diagnostics Group PLC

1. Table of contents

1. Table of contents 1
2. Purpose & scope 1
3. Responsibilities & safety precautions 1
4. Equipment & reagents 1
5. Definitions & abbreviations 2
6. Procedure 2
7. Appendices 6
8. Other related procedures & documents 6

2. Purpose & scope

To explain the adapted protocol for the analysis of dried blood specimens including dried blood spots samples dried onto Whatman 903 protein saver cards and samples collected onto filter paper using the Drawbridge OneDraw device using the Omega Diagnostics COVID-19 IgG ELISA test.

3. Responsibilities & safety precautions

4. Equipment & reagents

Equipment:

- Roller mixer
- Centrifuge
- Automated plate washer
- Plate reader capable of reading at 450 and 620nm
- Perkin Elmer DBS Puncher (for dried blood spot samples only)
- Anti-static gun
- Magnetic stirrer with flees
- pH meter
- Multi-channel repeater pipette with width-adjustment and pipette-tips suitable for transfer of 200ul of sample material.
- Standard repeater pipette with 5ml barrels.

Reagents:

- Omega Diagnostics COVID-19 IgG ELISA kit.
- Invitrogen Phosphate Buffered Saline tablets Product code: 003002.
- Corning™ 96-Well Clear Ultra Low Attachment Microplates. Product code: 10023683

- Deionised water (resistivity >18.2mΩ)
- 50 ml Falcon tubes

Quality Control:

Serum Low: Produce 50 ul aliquots of human serum from an individual with negative COVID-IgG status (as categorised by the Omega Diagnostics assay).

Serum High: Produce 50 ul aliquots of human serum from an individual with positive COVID-IgG status (as categorised by the Omega Diagnostics assay), preferably with a measured absorbance near to the cut-off value.

5. Definitions & abbreviations

Definition/Abbreviation	Description

6. Procedures:

6.1 Onedraw / DBS preparation

Reagents

Assay Buffer Preparation

N.B. Assay buffer is supplied in the Omega Diagnostics COVID-19 IgG ELISA kit.

Dilute stock assay buffer x15 with deionised water (e.g. 3 mls stock concentration assay buffer made up to 45 mls with deionised water). Store at +4°C for up to 6 months.

Phosphate Buffered Saline Preparation

Add 20 PBS tablets to a 2L bottle. Add 2 L deionised water. Mix on magnetic stirrer until tablets have completely dissolved. Check pH and adjust to pH 7.2 if required.

Dried Blood Spot sample preparation

NB. Sample stability for DBS samples has not yet been established. It is recommended that samples which can be analysed within 1 week of receipt are to be stored at room temperature. Samples which need to be stored for longer should be stored at -80°C. Each sample should be stored in a sealed zip-lock bag containing a desiccant pouch.

Elutions of DBS samples should be prepared on the day prior to analysis as an overnight incubation is required.

If DBS samples are stored frozen or chilled, allow to equilibrate to room temperature before removing from zip-lock bag.

Switch on DBS puncher

Select “TSH” protocol (this protocol is set-up to punch a single 3.2mm disc per well of a 96-well microtitre plate).

Use anti-static gun to remove static charge from a Corning™ 96-Well Clear Ultra Low Attachment Microplate and load onto the DBS puncher

Turn the DBS card face down and place a portion of the sample which is saturated with blood under the foot of the puncher.

Gently push down on the plate to initiate the punch. Record the sample ID and position on plate (e.g. A1).

The puncher will automatically move to the next well.

When punching is complete select “end punching” on DBS puncher and remove plate.

Using a repeater pipette Add 300ul of Assay buffer per well.

Seal plate and shake at 450 RPM overnight.

Onedraw Drawbridge sample preparation

NB. Sample stability for Drawbridge Onedraw samples has not yet been established. It is recommended that samples which can be analysed within 1 week of receipt are to be stored at room temperature. Samples which need to be stored for longer should be stored at -80 C. Each sample should be stored in a sealed zip-lock bag containing a desiccant pouch.

If Onedraw samples are stored frozen or chilled, allow to equilibrate to room temperature before removing from zip-lock bag.

Using tweezers, remove the strip for analysis and place into a labelled 2 ml tube.

Repeat for all samples to be analysed.

Using a repeater pipette Add 1.8 mL of PBS per tube.

Seal plate and shake at 650 RPM overnight.

Dilute the PBS eluent 1:3 in Assay Buffer, 200 ul PBS eluent + 600 ul assay buffer to produce assay dilution (equivalent to ~1:200 serum dilution). The assay dilution is suitable for COVID-IgG analysis only. The PBS elution is stored for future projects.

Store residual PBS eluent at -80 C.

Store assay dilution at -80 C.

6.2 Analysis

N.B. This protocol is to be used as a supplement to the Omega Diagnostics IFU and includes our in-house adaptation for compatibility with DBS / Onedraw elution samples.

Remove the ELISA kit from refrigerator storage.

Place the Conjugate, TMB substrate, stop, sample diluent concentrate, wash buffer concentrate and kit controls on a roller mixer and allow to equilibrate to room temperature prior to analysis.

Remove Serum L & H controls from -80 C storage and place on roller mixer. Allow to defrost before centrifuging at 4000rpm for 10 minutes. Dilute 1:200 in assay buffer in a labelled 15 mL tube. 30 ul serum + 6 ml assay buffer, these dilutions can be stored at +4 C for up to a week.

For DBS sample analysis: DBS sample elutions (see 6.1 for DBS preparation) can be analysed as per Omega Diagnostics protocol for 1:200 dilutions of human serum.

For Onedraw sample analysis: Onedraw sample “assay dilution” preparations (see 6.1) can be analysed as per Omega Diagnostics protocol for 1:200 dilutions of human serum.

Use a hard plastic 9x9 sample rack to rack controls + samples for analysis (apart from for any DBS samples which need to be added directly from the elution plate). Kit controls and blanks need to be transferred from primary containers to Sarstedt 2ml tubes (N.B. There needs to be sufficient volume to load plate and enough spaces to insert the pipette tip without the tube overflowing):

A1: BLK Sample Diluent
B1: Cut-off control

C1: Positive control (green solution)

Prepare the serum controls: 5µl diluted with 1ml sample diluent, add to the already racked controls:

D1: Serum L

E1: Serum H

Make sure that the lids of the tubes are labelled

Once the samples have defrosted , centrifuge for 10 minutes at 4000rpm:
Once spun transfer to the loading rack in a “column-wise” fashion so that the rack is loaded in the order that the plate will be filled.

Sample loading rack map

	1	2	3	4	5	6	7	8	9
A	BLANK	UNK-4	UNK-12	UNK-20	UNK-28	UNK-36			
B	CUT-OFF	UNK-5	UNK-13	UNK-21	UNK-29	UNK-37			
C	POS	UNK-6	UNK-14	UNK-22	UNK-30	UNK-38			
D	SERUM-L	UNK-7	UNK-15	UNK-23	UNK-31	UNK-39			
E	SERUM-H	UNK-8	UNK-16	UNK-24	UNK-32	BLANK			
F	UNK-1	UNK-9	UNK-17	UNK-25	UNK-33	CUT-OFF			
G	UNK-2	UNK-10	UNK-18	UNK-26	UNK-34	SERUM-L			
H	UNK-3	UNK-11	UNK-19	UNK-27	UNK-35	SERUM-H			
I									

Using the multichannel repeator pipette aspirate 200µL (with pre-step) and dispense 100µL per well; RETURN the excess in the tips back to the ORIGINAL tube.
Repeat with fresh tips
Include the following controls in the final column.:

F1: BLK Sample Diluent

E1: Cut-off control

G1: Serum L

H1: Serum H

Do not include the positive control in the end column.

Plate Map

	1	2	3	4	5	6	7	8	9	10	11	12
A	BLANK	BLANK	UNK-4	UNK-4	UNK-12	UNK-12	UNK-20	UNK-20	UNK-28	UNK-28	UNK-36	UNK-36
B	CUT-OFF	CUT-OFF	UNK-5	UNK-5	UNK-13	UNK-13	UNK-21	UNK-21	UNK-29	UNK-29	UNK-37	UNK-37
C	POS	POS	UNK-6	UNK-6	UNK-14	UNK-14	UNK-22	UNK-22	UNK-30	UNK-30	UNK-38	UNK-38
D	SERUM-L	SERUM-L	UNK-7	UNK-7	UNK-15	UNK-15	UNK-23	UNK-23	UNK-31	UNK-31	UNK-39	UNK-39
E	SERUM-H	SERUM-H	UNK-8	UNK-8	UNK-16	UNK-16	UNK-24	UNK-24	UNK-32	UNK-32	BLANK	BLANK
F	UNK-1	UNK-1	UNK-9	UNK-9	UNK-17	UNK-17	UNK-25	UNK-25	UNK-33	UNK-33	CUT-OFF	CUT-OFF
G	UNK-2	UNK-2	UNK-10	UNK-10	UNK-18	UNK-18	UNK-26	UNK-26	UNK-34	UNK-34	SERUM-L	SERUM-L
H	UNK-3	UNK-3	UNK-11	UNK-11	UNK-19	UNK-19	UNK-27	UNK-27	UNK-35	UNK-35	SERUM-H	SERUM-H

Once plate is loaded, follow steps in the Omega Diagnostics IFU for sample/conjugate/substrate incubations. Add stop solution as per IFU. Read plate on Thermo Multiskan.

7. Appendices

8. Other related procedures & documents
